# Characterization of Shiga Toxin Subtypes and Virulence Genes in Porcine Shiga Toxin-Producing *Escherichia coli*

**DOI:** 10.3389/fmicb.2016.00574

**Published:** 2016-04-21

**Authors:** Gian Marco Baranzoni, Pina M. Fratamico, Jayanthi Gangiredla, Isha Patel, Lori K. Bagi, Sabine Delannoy, Patrick Fach, Federica Boccia, Aniello Anastasio, Tiziana Pepe

**Affiliations:** ^1^Eastern Regional Research Center, United States Department of Agriculture – Agricultural Research ServiceWyndmoor, PA, USA; ^2^Center of Food Safety and Applied Nutrition, U.S. Food and Drug AdministrationLaurel, MD, USA; ^3^Food Safety Laboratory, University of Paris-Est, Anses, Maisons-AlfortFrance; ^4^Department of Veterinary Medicine and Animal Production, University of Naples Federico II, NaplesItaly

**Keywords:** *Escherichia coli*, STEC, swine, Shiga toxins variants, virulence genes

## Abstract

Similar to ruminants, swine have been shown to be a reservoir for Shiga toxin-producing *Escherichia coli* (STEC), and pork products have been linked with outbreaks associated with STEC O157 and O111:H-. STEC strains, isolated in a previous study from fecal samples of late-finisher pigs, belonged to a total of 56 serotypes, including O15:H27, O91:H14, and other serogroups previously associated with human illness. The isolates were tested by polymerase chain reaction (PCR) and a high-throughput real-time PCR system to determine the Shiga toxin (Stx) subtype and virulence-associated and putative virulence-associated genes they carried. Select STEC strains were further analyzed using a Minimal Signature *E. coli* Array Strip. As expected, *stx*_2e_ (81%) was the most common Stx variant, followed by *stx*_1a_ (14%), *stx*_2d_ (3%), and *stx*_1c_ (1%). The STEC serogroups that carried *stx*_2d_ were O15:H27, O159:H16 and O159:H-. Similar to *stx*_2a_ and *stx*_2c_, the *stx*_2d_ variant is associated with development of hemorrhagic colitis and hemolytic uremic syndrome, and reports on the presence of this variant in STEC strains isolated from swine are lacking. Moreover, the genes encoding heat stable toxin (*estIa*) and enteroaggregative *E. coli* heat stable enterotoxin-1 (*astA*) were commonly found in 50 and 44% of isolates, respectively. The hemolysin genes, *hlyA* and *ehxA*, were both detected in 7% of the swine STEC strains. Although the *eae* gene was not found, other genes involved in host cell adhesion, including *lpfA_O113_* and *paa* were detected in more than 50% of swine STEC strains, and a number of strains also carried *iha, lpfA_O26_, lpfA_O157_, fedA, orfA*, and *orfB*. The present work provides new insights on the distribution of virulence factors among swine STEC strains and shows that swine may carry Stx1a-, Stx2e-, or Stx2d-producing *E. coli* with virulence gene profiles associated with human infections.

## Introduction

Shiga Toxin-producing *Escherichia coli* (STEC) are food-borne pathogens responsible for outbreaks and serious illness including hemorrhagic colitis (HC) and hemolytic uremic syndrome (HUS). STEC O157:H7 is the serotype that has most often been associated with outbreaks and severe forms of diarrhea; however, recently a number of non-O157 STEC serogroups that cause similar illnesses have emerged (Gould et al., 2013). Cattle and other ruminants are important reservoirs of STEC; infection is asymptomatic, and they can carry the pathogens for long periods of time. Similarly, healthy swine may shed STEC, as demonstrated by several studies in which STEC were detected and isolated from swine fecal samples (Tseng et al., 2014b). Many of the investigations focused on serotype O157:H7; however, some studies also tested for non-O157 STEC serogroups and identified serogroups previously associated with human cases of illness (Fratamico et al., 2004; Kaufmann et al., 2006; Tseng et al., 2014b). The possibility that swine can transmit pathogenic STEC to humans is supported by a few outbreaks linked to the consumption of pork products contaminated with STEC O157:H7, O157:NM, and O111:H- (Tseng et al., 2014b).

Shiga toxins (Stx) are divided in two major antigenic forms: Stx1 and Stx2. Variants for Stx1 and Stx2 are grouped in three (Stx1a, Stx1c, Stx1d) and seven (Stx2a, Stx2b, Stx2c, Stx2d, Stx2e, Stx2f, and Stx2g) subtypes, respectively (Scheutz et al., 2012). Although Stx1a has been linked to human illness, STEC that produce subtypes Stx2a, Stx2c, and Stx2d are more often associated with the development of HC and HUS (Friedrich et al., 2002; Melton-Celsa, 2014). *In vitro* studies in two different cell lines showed that Stx2a and Stx2d were more potent than Stx2b and Stx2c. These results were also confirmed by experimentation in mice showing a significantly higher potency of Stx2a and Stx2d than Stx1, Stx2b, and Stx2c (Fuller et al., 2011). Stx variants are not homogeneously distributed among the STEC population and certain variants are frequently detected in association with different animals (Martin and Beutin, 2011; Hofer et al., 2012; Fuente et al., 2015). Swine STEC strains commonly produce Stx2e (Fratamico et al., 2004; Meng et al., 2014; Tseng et al., 2015), which may cause edema disease in weaned pigs, often leading to ataxia and death. Stx2e-producing *Escherichia coli*, do not represent a particular threat for humans (Friedrich et al., 2002; Tseng et al., 2014b). Nevertheless, STEC carrying the *stx*_2e_ gene have been isolated from human cases with mild diarrhea (Muniesa et al., 2000; Friedrich et al., 2002; Beutin et al., 2004; Sonntag et al., 2005) and from two patients with HUS (Thomas et al., 1994; Fasel et al., 2014). The severe outcome of the first HUS case was probably due to a co-infection with another STEC strain (Thomas et al., 1994), while the second patient with HUS was described as having a very weak immune system (Fasel et al., 2014). Besides Stx2e, there is a lack of information on the presence of other Stx subtypes in STEC strains isolated from swine.

The production of Stx is necessary to provoke HUS; however, other virulence factors are also important in causing illness. These include genes involved in cell adhesion, proteases, and toxins, as well as other putative virulence factors. The presence of specific combinations of virulence factors may determine the risk of developing severe symptoms. The *eae* gene, found on the locus of enterocyte effacement (LEE), encodes intimin, which is an adhesin involved in gut colonization. LEE-positive STEC are expected to provoke HUS or HC more frequently than LEE-negative STEC (Ethelberg et al., 2004; Toma et al., 2004; Luna-Gierke et al., 2014). Nevertheless, cases of HUS provoked by LEE-negative STEC have been reported (Karmali et al., 1985; Paton et al., 1999; Bielaszewska et al., 2009), including a large outbreak in 2011 in Europe caused by an enteroaggregative *E. coli* that acquired the *stx*_2a_ gene, and it possessed a combination of virulence genes increasing its virulence (Boisen et al., 2015). This suggests that LEE is not essential in the development of severe symptoms, and other genes involved in adherence may also be important. Many adherence gene candidates, including *eibG*, *lpfA*, *saa*, and *sab* have been identified in STEC (Croxen et al., 2013). Nevertheless, mechanisms for attachment of LEE-negative STEC to the intestinal epithelium have not been studied as extensively as attachment of LEE-positive STEC.

In 2000, one objective of the U.S. Department of Agriculture’s Animal and Plant Health Inspection Service National Animal Health Monitoring System (NAHMS) Swine 2000 study was to determine the prevalence of STEC in swine. Fecal samples were from states with the highest production of swine in the U.S. (U.S. Department of Agriculture, 2001). As a result of this work, 219 STEC isolates were recovered and characterized (Fratamico et al., 2004, 2008). Since this work was conducted, the knowledge of the importance of non-O157 STEC in human illness has increased, and there is a need to develop a model for molecular risk assessment associated with STEC. Knowledge of the virulence gene combinations that distinguish highly pathogenic *E. coli* from less virulent strains remains unclear, particularly for LEE-negative STEC (Beutin and Fach, 2014). Additionally, new virulence-associated and putative virulence-associated factors are being identified (Coombes et al., 2008; Brandt et al., 2011; Bugarel et al., 2011). The aim of the present study was to characterize STEC recovered from swine, belonging to a variety of serotypes to determine their Stx subtype and virulence gene profiles to understand their virulence potential.

## Materials and Methods

### Bacterial Strains

Swine STEC strains were isolated and serotyped during the NAHMS swine 2000 study (NAHMS 2000) as described by Fratamico et al. (2004). Briefly, fresh swine feces were recovered from the pen floor of swine operations from the main pork-producing states in U.S. A total of 687 swine fecal samples were enriched using tryptic soy broth (TSB) and screened for the presence of *stx*_1_ and *stx*_2_ by polymerase chain reaction (PCR). Positive samples were plated onto Luria-Bertani agar, and *stx*_1_- and *stx*_2_-positive colonies were detected following DNA hybridization and confirmed by PCR. Two hundred and nineteen STEC strains were serotyped and frozen in TSB with 20% of glycerol. From this collection, 181 STEC strains were used in this study and maintained on tryptic soy agar plates or TSB as working stock cultures.

Besides the NAHMS swine isolates, three STEC O91 strains from our collection were also used for comparison. STEC O91:H14 (strains 2.4111 and 2.4114) were isolated from ground beef while STEC O91:H21 (strain B2F1) was isolated from a case of HUS (Ito et al., 1990).

### Identification of Shiga-toxin Subtypes

DNA extraction and PCR assays to identify *stx* subtypes and *stx* partial sequences were performed according to Scheutz et al. (2012) using a ProFlex PCR system (Thermo Fisher, Waltham, MA, USA) with slight modifications. TaqMan Environmental Master Mix 2.0 (Thermo Fisher) was used, and the annealing temperature was raised to 65°C when cross-reaction was observed, as suggested by the authors (Scheutz et al., 2012). Gel electrophoresis was performed using 1.5% UltraPure Agarose (Invitrogen, Carlsbad, CA, USA) gel with 0.5X GelRed (Phenix Research Products, Candler, NC, USA) in 1X Tris-acetate-EDTA buffer at 100 V for 1 h. One microliter of amplified DNA was analyzed by agarose gel electrophoresis and visualized using an AlphaImager gel documentation system (Alpha Innotech, San Leandro, CA, USA).

Polymerase chain reaction products for sequencing were cleaned with Agencourt AMPure XP (Beckman Coulter, Brea, CA, USA), and 1.2 μl were amplified in a reaction consisting of 7 μl of 2.5X buffer, 1 μl of 3.2 μM primer stx2-F4 or stx2-R1 (Scheutz et al., 2012), 1 μl of Big Dye Terminator (Applied Biosystems), and 10 μl of nuclease-free water. Thermocycling conditions consisted of 30 cycles of 95°C for 10 s, 55°C for 5 s and 60°C for 4 min. The sequencing reaction products were then purified and sequenced using Agencourt CleanSEQ (Beckman Coulter) and 3730 DNA Analyzer (Applied Biosystems), respectively. The sequences were manually curated using Sequencher v5.2.3 (Gene Code Corporation, Ann Arbor, MI, USA), run in VirulenceFinder 1.5 (Joensen et al., 2014), and blasted against the NCBI database^1^. The nucleotide sequences were deposited in the GenBank nucleotide sequence database under the following accession numbers: strain 306, KU682619; strain 308, KU682620; strain 326, KU682621; strain 341, KU682622; strain 360, KU682623, and strain 500, KU682624.

### High-throughput Real-time PCR Assay and Testing for Hemolysis

DNA was extracted from the swine isolates using the PrepMan Ultra Sample Preparation Reagent (Thermo Fisher) according to the manufacturer’s instructions. The high-throughput real-time PCR (hrPCR) assay was carried out using the BioMark real-time PCR system (Fluidigm, San Francisco, CA, USA), targeting 67 virulence-associated and putative virulence-associated genes, 14 O-group-associated genes (O26, O45, O55, O91, O103, O104, O111, O113, O118, O121, O128, O145, O146, and O157) and 11 H-group-associated genes (H2, H4, H7, H8, H11, H16, H19, H21, H25, H28, and H32). Primers were designed in several studies (Perelle et al., 2003; Fratamico et al., 2008; Bugarel et al., 2010, 2011; Delannoy et al., 2013) and summarized by Tseng et al. (2014a). Reagents for DNA amplification and thermal cycling conditions were previously reported (Tseng et al., 2014a). Swine STEC strains positive to *ehxA* and *hlyA* genes were tested for hemolysis by plating onto SHIBAM agar (Hardy Diagnostic, Santa Maria, CA, USA).

### FDA Minimal Signature *E. coli* Array

Swine Stx2d-producing *E. coli* and non-Stx2e STEC belonging to a serotype associated with human disease were further analyzed using the Minimal Signature *E. coli* Array Strip (FDA-ECID; Affymetrix, Santa Clara, CA, USA). Genomic DNA was isolated and concentrated using the DNeasy Tissue Kit (QIAgen Inc., Valencia, CA, USA) and SC100 Speedvac Concentrator (Savant Instruments, Inc. Holbrook, NY, USA), respectively. Two micrograms of DNA were tested using the FDA-ECID array as described in detail by Lacher et al. (2014). Robust multi-array average summarized probe intensity data were analyzed using R-Bioconductor software v3.1.2 and affy package with parameters defined by Lacher et al. (2014). The Hierarchical clustering was done using overview function in MADE4 package that uses average linkage cluster analysis with a correlation metric distance (Culhane et al., 2005; Culhane and Thioulouse, 2006).

## Results

### Swine STEC Serotypes

**FIGURE 1:**
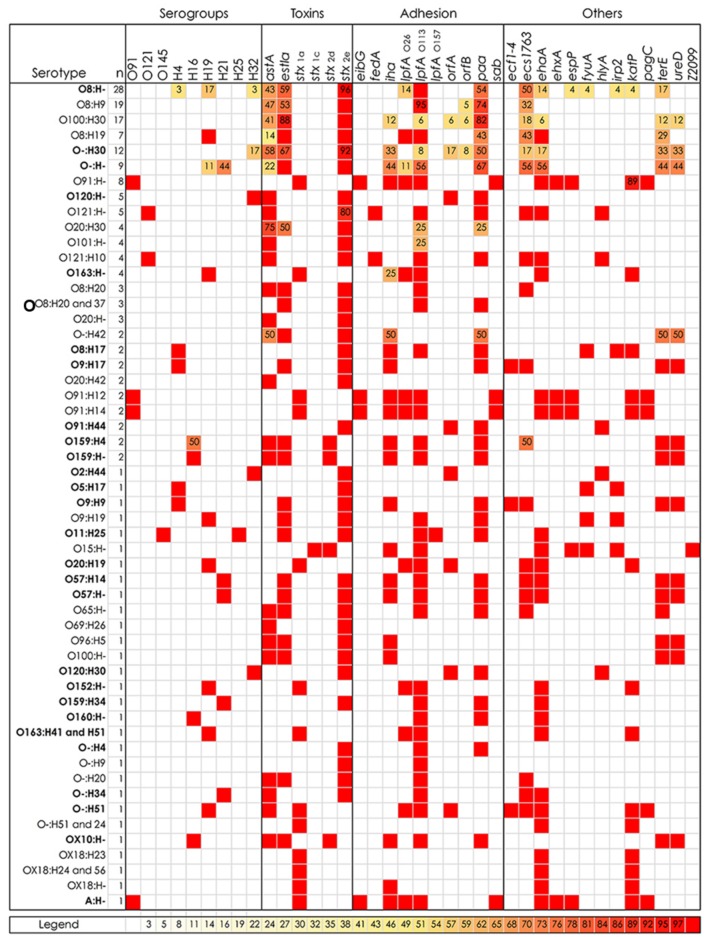
**Distribution of virulence factors and serogroup markers of Shiga toxin-producing *Escherichia coli* (STEC) isolated from swine feces.** Percentage of positive STEC strains within each serotype is reported in cells with numbers and a three-color scale. White cells and red cells correspond to 0 and 100%, respectively. Serotype: A, autoagglutination; O-, O non-typeable; H-, H non-typeable; bolded, hrPCR results and traditional serotype were different. All the swine STEC analyzed with hrPCR assay resulted negative for: *bfp, cdtI, cdtIII, cnf2, eae, eae*_α_, *eae*_β_, *eae*_𝜀_, *eae*_γ_, *eae*_𝜃_, *ecs1822, efa*1, *elt, ent/espL2, epeA, espK, espM1, espM2, espN, espO1-1, espV, espX7, etpD, fasA, fimF41a, nleA, nleB, nleE, nleF, nleG5, nleG6-2, nleH1-2, sfp, saa, stcE, stx*_1d_, *stx*_2a_, *stx*_2b_, *stx*_2c_, *stx*_2f_, *stx*_2g_, *subAB, toxB, Z2096, Z2098*, O26, O45, O55, O103, O104, O111, O113, O118, O128, O146, O157, H2, H7, H8, H11, and H28 (data not reported in the Figure).

All of the strains had been previously serotyped at the *E. coli* Reference Center at the Pennsylvania State University (University Park, PA, USA). In addition, many O-group- and H-group- specific targets were included in the hrPCR assay. Several discrepancies were found and serotypes that did not match with the traditional serotyping are indicated in bold in **Figure 1**. Selected swine STEC strains were also analyzed using the FDA-ECID microarray, and the resulting serotypes were in agreement with the hrPCR. Moreover, the grouping within the phylogenetic tree was consistent with the serotypes proposed by the FDA-ECID microarray (**Table 1**).

**Table 1:** Serotypes and phylogenetic tree of select swine STEC strains analyzed using the FDA-ECID array.

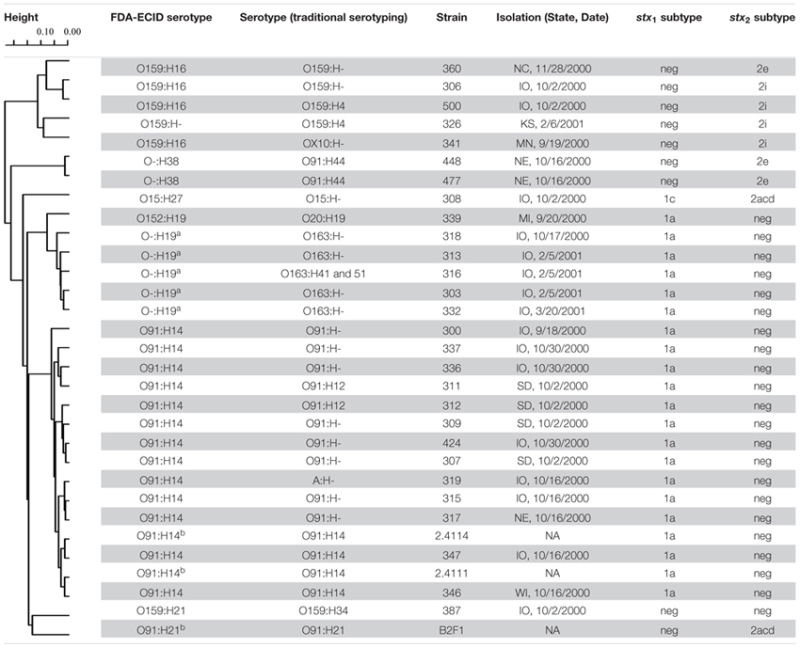

*A, autoagglutination; O-, O non-typeable; H-, H non-typeable; A; neg, negative results; NA, not available.*

*^a^The FDA-ECID does not include probes for O163 markers.*

*^b^Swine STEC isolates were analyzed together with two strains of *E. coli* O91:H14 and *E. coli* O91:H21 for comparison.*

### Shiga-toxin Subtype Characterization

The swine STEC strains were analyzed by singleplex and multiplex PCR assays to determine their Shiga-toxin subtype. Stx-encoding genes were carried by 177/181 (99.8%) of the tested isolates. Four strains previously identified as STEC likely lost the Stx genes due to loss of Stx-encoding phages, as has been shown by other investigators (Joris et al., 2011) since PCR results were negative for any of the subtypes. *stx*_1_ or *stx*_2_ genes were carried by 25 and 151 strains, respectively. Stx subtype analysis revealed that the 25 *stx*_1_-positive strains carried the *stx*_1a_ subtype. Among the 151 *stx*_2_-positive strains, 146 and 5 isolates carried *stx*_2e_ and *stx*_2d_, respectively. STEC strain 308 was the only isolate that carried both *stx*_1_ and *stx*_2_, subtypes *stx*_1c_ and *stx*_2d_, respectively. Stx subtypes divided by STEC serotype are reported in **Figure 1**. Strains carrying Stx subtypes *stx*_1d_, *stx*_2a_, *stx*_2b_, *stx*_2c_, *stx*_2f_, and *stx*_2g_ were not identified. Selected swine strains were analyzed using the FDA-ECID microarray and results of Stx subtypes are reported in **Table 1**. Nucleotide sequencing of *stx*_2_ was carried out from STEC strains that were *stx*_2d_ positive by PCR. The STEC strain 308 *stx*_2_ sequence showed 100% identity to a portion of *stx*_2d_ subunit B (AF479829) using VirulenceFinder. When blasted against the NCBI database, the STEC strain 308 *stx*_2_ partial sequence matched EF441621 (*stx*_2d_) with 100% identity and no gaps. The *stx*_2_ partial sequences of STEC strains 306, 326, 341, 360, and 500 were identical. VirulenceFinder results showed 100% identity with a portion of *stx*_2d_, subunit B (DQ059012). The most similar sequence present in the NCBI database was KC339670 (*stx*_2e_) with an identity on 99%.

### Distribution of Virulence-associated Genes among the Swine STEC Collection

Genomic DNA extracted from the swine STEC strains was analyzed using hrPCR. Genes encoding the enteroaggregative *E. coli* heat-stable enterotoxin 1 (*astA*) and the heat-stable enterotoxin (*estIa*) were detected in 79 (44%) and 91 (50%) of the isolates, respectively. Toxins and cytotoxic factors encoded by *cdtI*, *cdtIII*, *elt, ent*/*espL2, cnf2*, and *subAB* were not detected. Regarding cytolysins, enterohemolysin (*ehxA*) and α-hemolysin (*hlyA*) encoding genes were found non-simultaneously in 13 (7%) of the swine STEC strains each. These strains were also hemolytic when plated onto SHIBAM plates.

None of the isolated swine STEC strains carried the intimin-encoding gene, *eae*, effector genes involved in the type III secretion system (*espK*, *espM1*, *espM2*, *espN*, *espO1-1*, *espV*, *espX7*, *nleA*, *nleB*, *nleE*, *nleF*, *nleG5*, *nleG6-2*, and *nleH1-2*) or the type II secretion system effector (*etpD*). Other genes encoding factors involved in adhesion and colonization to the host intestine were also investigated. Among these, the most prevalent were *lpfA*_O113_, *paa*, *ihA*, and *lpfA*_O26_ present in 116 (64%), 98 (54%), 41 (23%), and 33 (18%) isolates, respectively. Genes *orfA*, *orfB*, and *fedA* were detected in less than 8% of the isolates. While *bfp*, *efa1*, *fasA*, *fim*_F41a_, *saa*, and *toxB* were not found, one swine STEC strain carried *lpfA*_O157_. Three autotransporter protein genes *ehaA, espP*, and *sab* were found in 60 (33%) 13 (7%), and 15 (8%) isolates.

Other gene targets were also included in the high-throughput real-time PCR assay. Positive results were obtained for the *ecs1763*, *terE, katP*, and *ureD* genes in 56 (31%), 31 (17%), 27 (15%), and 23 (13%) of isolates, respectively. While less than 8% were positive for *pagC eibG*, *irp2*, *fyuA*, *ecf1*, *ecf2*, *ecf3*, *ecf4*, and *Z2099.* None of the swine STEC strains carried *ecs1822*, *epeA*, *sfp*, *stcE*, *Z2096*, or *Z2098*.

## Discussion

It is well-known that swine shed a variety of STEC serogroups, which may be carried along the food production chain. Most of the STEC isolated from these animals have adapted to the swine host and seem to have low potential to infect humans. Nevertheless, outbreaks associated with pork products have occurred (Meng et al., 2014; Tseng et al., 2014b). The sampling area covered by the NAHMS swine 2000 study was large, covering all the main pork-producing States (Fratamico et al., 2004). A subset of 181 STEC strains were analyzed and their pathogenic potential was assessed by detection of virulence and putative virulence factors.

The *stx* subtypes carried by the swine STEC were identified, and the majority of the isolates carried *stx*_2e_ (81%), which was consistent with the data reported by Fratamico et al. (2004). The second most prevalent subtype was *stx*_1a_ (14%), followed by *stx*_2d_ (3%), and *stx*_1c_ (1%). Stx2d is a potent toxin, and infection with strains carrying this subtype can lead to severe symptoms such as HC and HUS in humans (Melton-Celsa, 2014). Besides the Stx genes, the thermostable enterotoxin genes, *astA* and/or *estIa*, genes were found in ∼71% of the isolates. Thermostable enterotoxins are usually carried by enterotoxigenic *E. coli*, which are the major pathogens responsible for traveler’s diarrhea. Twenty-two percent of the swine STEC strains were positive for both genes. The exotoxins HlyA (α-hemolysin) and EhxA (enterohemolysin) produce pores in the cytoplasmic membranes of eukaryotic cells causing their death. Their role in STEC pathogenesis is still not clear; HlyA may increase the virulence of extraintestinal pathogenic *E. coli* and, in the case of EhxA, a correlation between *ehxA*-positive STEC and development of severe symptoms in humans has been observed (Karch and Bielaszewska, 2001; Mainil, 2013). Thirteen isolates carried the *hlyA* gene. Nine of them belonged to serotypes O121:H- or O121:H10, presenting a virulence gene profile typical of strains associated with edema disease in swine due to the presence of *stx*_2e_, *hlyA* and *fedA* (Tseng et al., 2014b). The *ehxA* gene is commonly found in STEC. From 40 to 77% of strains collected from patients, food, and cattle carry this gene (Karch and Bielaszewska, 2001; Slanec et al., 2009; Bosilevac and Koohmaraie, 2011; Feng, 2014). Swine isolates appear to carry *ehxA* less frequently (Meng et al., 2014; Tseng et al., 2014a), and this observation is in agreement with our study where only 7% of the isolates was *ehxA* positive.

All of the swine STEC strains were LEE-negative. Although the adhesion mechanisms of LEE-negative STEC are not well characterized, several factors have been described to play an important role in adhesion to the intestinal epithelium. The long polar fimbriae gene *lpfA_O113_* was identified in STEC O113:H21 (Doughty et al., 2002). These investigators demonstrated that the removal of *lpfA_O113_* reduces the bacterial capacity to adhere to epithelial cells. Similar *lpfA* genes were found in *E. coli* O157 and O26 (Hayashi et al., 2001; Toma et al., 2004). Another bacterial adherence-conferring gene is the iron-regulated gene A homolog adhesin *iha*. Similarly to *lpfA_O113_*, the *iha* gene is commonly found in STEC strains associated with human cases of HUS (Newton et al., 2009; Galli et al., 2010). Nevertheless, non-pathogenic *E. coli* can also carry *lpfA_O113_* and *iha*, suggesting that the presence of these genes is insufficient to establish an infection (Toma et al., 2004). Over 80% of the strains analyzed in this study carried *lpfA*_O26_, *lpfA_O113_*, or *lpfA_O157_*; while *iha* was found in almost one quarter of swine isolates. *iha*-positive STEC were also described in a longitudinal study of two Midwestern U.S. pork production sites (Tseng et al., 2014a, 2015). On the contrary, none of the swine STEC strains collected in another interesting study in China carried *iha* (Meng et al., 2014). The second most prevalent adhesion factor found in this dataset was the porcine attaching and effacing-associated adhesin, *paa*, which is associated with neonatal post-weaning diarrhea in pigs (An et al., 1999). In addition, a few strains carried *orfA* and *orfB*, which encode for adhesins involved in diffuse adherence (Charbonneau et al., 2006).

Autotransporter proteins have a peculiar structure that allows them to move through the membrane system and execute their function outside the bacterial cell. The genes *ehaA* and *sab* were discovered in O157:H7 strain EDL933 and LEE-negative O113:H21, respectively. They encode for two different autotransporter proteins that contribute to adhesion and biofilm formation (Wells et al., 2008; Herold et al., 2009). Together with LEE genes, *iha* and *ehaA* are highly expressed in the intestines of pigs presenting attaching and effacing lesions (Liu et al., 2015). While the *ehaA* gene was present in over 30% of the swine isolates, *sab* was carried by 13 STEC strains only belonging to O-group O91.

As stated above, 12 to 18% of the isolates were positive for *katP, ureD*, and *terE*. The genes *katP* and *ureD* encode for a catalase/peroxidase and urease transporter, respectively. Their role in *E. coli* pathogenesis is unclear; however, they appear to be prevalent in diarrheagenic *E. coli* (Dorothea et al., 2006; Delannoy et al., 2013). The gene *terE* is a component of the *ter* cluster, which confers tellurite resistance (Orth et al., 2007). The *ecs1763* and *ecs1822* genes have been proposed to be novel markers for enterohemorrhagic *E. coli*. Their function is unknown, and they were shown to be shared by a clonal group of enterohemorrhagic *E. coli* that includes O26, O111, and O118 (Abu-Ali et al., 2009). Tseng et al. (2014a) observed that *ecs1763* is frequently found in swine STEC, which was confirmed by the present study where 31% of the isolates carried *ecs1763*. *ecs1822* was absent in all the tested strains.

Traditional serotyping of *E. coli* is time consuming, and cross-reactions among antisera often occur. Based on the hrPCR and FDA-ECID results, 71 strains present in this collection belonged to serotypes O8:H9, O8:H19, O8:H-, O15:H27, O20:H-, O91:H14, O101:H-, O121:H-, O145:H25, O159:H21, and O163:H19 that were previously isolated from human patients (Blanco et al., 1992; Beutin and Fach, 2014). Serotypes O8:H19, O15:H27, O145:H25, and O163:H19 have also been associated with cases of HUS (Prager et al., 2005; Bielaszewska et al., 2006; Galli et al., 2010). All of the strains belonging to the serotypes O8:H9, O8:H19, O8:H-, O20:H-, O101:H-, O121:H-, O145:H25, and O159:H21 analyzed in this study carried *stx*_2e_, which is a subtype that is generally not associated with STEC that cause serious human illness. Human infections linked to Stx2e-producing *E. coli* generally cause asymptomatic infections or mild diarrhea (Tseng et al., 2014b). The work of Sonntag et al. (2005) reported that human Stx2e-producing *E. coli* carry different virulence factors compared to swine Stx2e-producing *E. coli* associated with edema disease. They also detected *fyuA* and *irp2* genes in five strains isolated from humans. These genes are included in the high-pathogenicity island (HPI), which is involved in the iron metabolism of *Yersinia*. Mouse models showed that the HPI increases *E. coli* virulence in extraintestinal infections (Schubert et al., 2002). The hrPCR results revealed that some swine STEC strains belonged to the same serotypes as human Stx2e-producing *E. coli* (O8:H19 and O8:H-) reported by Sonntag et al. (2005). STEC O8:H19 and STEC O8:H- also carried markers for the HPI. Moreover, their virulence gene profiles included adhesins (*lpfA_O26_*, *lpfA_O113_*, *paa*) and enterotoxins (*astA* and *estIa*), which suggest that they can potentially provoke mild diarrhea in humans. The HPI genes *fyuA* and *irp2* were also found in Stx2e-producing *E. coli* belonging to serotypes O5:H4 and O8:H4 (**Table 1**).

Shiga toxin-producing *Escherichia coli* strain 308 was re-typed as O15:H27 using the FDA-ECID array and was found to have the same *stx*_2d_ sequence as *E. coli* O15:H27 (strain 88-1509) in the STEC isolate database at Michigan State University^2^. *E. coli* strain 88-1509 was collected in 1988 from a human case of HC and HUS in Canada. Other strains belonging to serotype O15:H27 have been isolated from human and cattle feces, and from meat sources (Piérard et al., 1997; Woodward et al., 2002; Bosilevac et al., 2007; Galli et al., 2010). The LEE-negative swine STEC O15:H27 has a virulence gene profile consisting of *stx*_1c_, *stx*_2d_, *ehaA*, *espP*, *fyuA*, *ihA, irp2*, *lpfA_O113_*, and *Z2099*. The relevance of some of these genes was mentioned above. *E. coli* secreted protein P (EspP) is an autotransporter protein with serine protease activity, and is used by the bacteria to impair the complement response of the host (Orth et al., 2010). Recently, In et al. (2013) reported that EspP boosts macropinocytosis in the intestinal epithelium increasing Stx uptake. The open reading frame *Z2099* is highly prevalent in typical and emerging enterohemorrhagic *E. coli*, while it is significantly less prevalent in non-pathogenic *E. coli* (Delannoy et al., 2013).

Six of the swine STEC strains carried *stx*_2d_ according to PCR and VirulenceFinder results, and they belonged to serotypes O159:H-, O159:H4, and OX10:H-. DebRoy et al. (2016) reported that serological cross-reactions between the O159 and OX10 O-groups often occur and that the nucleotide sequences of O159 and OX10 O-antigen gene clusters are almost identical. Based on the FDA-ECID analysis, the strains 306, 360, 341, and 500 were re-typed as O159:H16; while the strain 326 was re-typed as O159:H- (**Table 1**). STEC belonging to O-group O159 rarely infect humans (Brooks et al., 2005; Gould et al., 2013). STEC O159:H16 and O159:H- have been isolated only from swine samples, such as feces and carcasses (DesRosiers et al., 2001; Kaufmann et al., 2006; Meng et al., 2014). Stx subtype analysis of these strains often gives ambiguous results (Kaufmann et al., 2006; Meng et al., 2014). In this work, STEC O159:H16 and O159:H- were positive for *stx*_2d_ when tested by PCR; however, they were positive for *stx*_2e_ or *stx*_2i_ using the FDA-ECID array. Note that probes of the FDA-ECID array corresponding to *stx*_2i_ were designed using the Stx sequences AM904726 and FN252457 (Patel et al., 2016) that belong to the *stx*_2e_ subtype according to Scheutz et al. (2012). The product obtained from partial sequencing of *stx*_2_ was 99% identical to the sequence KC339670 when blasted against the NCBI database. KC339670 is a complete *stx*_2_ sequence belonging to a STEC O159:H16 strain isolated from swine in China. After a neighbor-joining cluster analysis of the sequence, Meng et al. (2014) concluded that KC339670 represented a new variant of *stx*_2e_. Further investigations using cell lines and animal models are needed to understand the virulence potential of this Stx2 variant. Another STEC O159 was detected in this collection. It belonged to the H21 H-group and was positioned distantly from the clade of O159:H16 and O159:H- (**Table 1**). This strain was positive for *stx*_2e_ only by PCR. *E. coli* belonging to serotype O159:H21 was isolated in 1983 during a small outbreak of diarrhea involving newborn children in Spain (Blanco et al., 1992), and no other infections associated with serotype O159:H21 have been reported.

Locus of enterocyte effacement-negative STEC belonging to O-group O91 are frequently associated with adult human infections with symptoms ranging from mild diarrhea to HC and HUS. The main serotypes are O91:H14 and O91:H21, and the latter is usually linked with development of severe symptoms (Bielaszewska et al., 2009). Human STEC O91:H14 and O91:H21 isolates carried mainly *stx*_1_ and *stx*_2d_, respectively (Prager et al., 2005; Bielaszewska et al., 2009; Galli et al., 2010). These STEC have been isolated from food samples derived from bovine, swine, and ovine origin, and from both domestic and wild animals (Martin and Beutin, 2011; Ju et al., 2012). From the NAHMS swine 2000 study, 15 strains belonging to serotypes O91:H12, O91:H14, O91:H44, and O91:H- were isolated from fresh fecal samples collected from four different states (Fratamico et al., 2004). Eight of these strains were re-typed as O91:H14, while STEC O91:H44 strains 448 and 477 did not belong to the O91 O-group based on FDA-ECID and hrPCR results (**Figure 1**; **Table 1**). STEC strain 319 that carried an identical virulence gene profile to other O91:H14 strains was also re-typed as O91:H14 by FDA-ECID array (**Table 1**). According to the phylogenetic tree in **Table 1**, the clade of STEC O91:H14 strains is well separated from the STEC O91:H21 strain B2F1 isolated from a human case of HUS. Interestingly, two O91:H14 strains were more closely related to two STEC O91:H14 strains isolated from ground beef samples than the other swine STEC O91:H14 strains. Despite the fact that one strain was *katP*-negative, all 13 STEC O91:H14 strains presented a conserved virulence gene profile (*ehaA, ehxA, eibG, espP, ihaA, katP, lpfA_O26_, lpfA_O113_, pagC, sab*, and *stx*_1a_), which is very similar to profiles of strains from human clinical samples (Prager et al., 2005; Bielaszewska et al., 2009). Similar to *ihaA, lpfA_O26_*, and *lpfA_O113_*, the proteins encoded by the genes *eibG* and *sab* are involved in host gut colonization. The *E. coli* immunoglobulin-binding protein encoded by *eibG* binds human immunoglobulin G and immunoglobulin A, and contributes to epithelial host cell adhesion (Lu et al., 2006), and *sab* is a gene encoding for an autotransporter protein involved in biofilm formation and found in a pathogenic LEE-negative STEC (Herold et al., 2009). Lastly, the *pagC* gene encodes for an outer membrane protein present in different Enterobacteriaceae that contributes to serum resistance (Nishio et al., 2005).

Human infections caused by STEC O163:H19 are rare (Brooks et al., 2005; Gould et al., 2013). However, Stx2-producing *E. coli* O163:H19 provoked sporadic cases of HUS (Prager et al., 2005) and have been found associated with cattle and produce (Woodward et al., 2002; Galli et al., 2010; Feng, 2014). In this work, five strains of STEC O163:H- or O163:H41/H51 were re-typed as O163:H19. They all carried *stx*_1a_ similar to the Stx1-producing *E. coli* O163:H19 strain isolated from swine by DesRosiers et al. (2001).

STEC O20:H19 is associated with human cases of HUS (Galli et al., 2010), and one strain belonging to this serotype was isolated in the NAHMS study (Fratamico et al., 2004). However, this same strain was re-analyzed using the FDA-ECID array, and it was re-typed as O152:H19, which is not known to be a human pathogen.

## Conclusion

Using state-of-the-art DNA-based techniques, this study provides new insights on the distribution of virulence factors in a heterogeneous collection of STEC isolated from the major pork-producing states of the U.S. Stx2e-producing *E. coli* known to provoke mild diarrhea in humans carried different virulence factors than Stx2e-producing *E. coli* associated with edema disease in pigs; this finding suggests that Stx2e-producing *E. coli* that cause human illnesses may not have a swine origin (Sonntag et al., 2005). In our work, STEC strains carrying *stx*_2e_ belonging to the same serotype and having similar virulence gene profiles as Stx2e-producing *E. coli* isolated from humans were identified. Additionally, the majority of Stx2e-producing *E. coli* carried thermostable enterotoxin genes usually found in enterotoxigenic *E. coli*.

This work suggests that STEC, including serotypes O15:H27 and O91:H14 that have been associated with human illness and are found in multiple hosts or environments, could also be carried by swine. Interestingly, a strain of O15:H27 found to carry *stx*_2d_ and other virulence genes may have the potential to produce severe symptoms in humans. Moreover, STEC O91:H14 strains presented a virulence gene profile very similar to profiles found in human isolates.

## Author Contributions

GMB and PF. designed research; GMB, LKB, SD, PF, FB, AA, and TP performed research; GMB, JG, and IP analyzed data; GMB and PF wrote the paper.

## Disclaimer

Mention of trade names or commercial products is solely for the purpose of providing specific information and does not imply recommendation or endorsement by the U.S. Department of Agriculture.

## Conflict of Interest Statement

The authors declare that the research was conducted in the absence of any commercial or financial relationships that could be construed as a potential conflict of interest.

## Footnotes

1 http://blast.ncbi.nlm.nih.gov/Blast.cgi

2 http://shigatox.net/new/database.html
